# CHH demethylation in the *ZmGST2* promoter enhances maize drought tolerance by regulating ROS scavenging and root growth

**DOI:** 10.1186/s12870-025-07012-9

**Published:** 2025-08-18

**Authors:** Xiaocui Yan, Mengjie Zhang, Yuan Zhong, Tinashe Zenda, Songtao Liu, Anyi Dong, Mengyu Kou, Jialong Liu, Nan Wang, Huijun Duan

**Affiliations:** 1 Present Address: State Key Laboratory of North China Crop Improvement and Regulation, North China Key Laboratory for Crop Germplasm Resources of Education Ministry, Hebei Agricultural University, Baoding, Hebei 071001 China; 2 Crop Science Department, Faculty of Plant and Animal Sciences and Technology, Marondera University of Agricultural Sciences and Technology, P. O. Box, 35, Marondera, Zimbabwe; 3 College of Agriculture and Forestry, Hebei North University, Zhangjiakou, 075000 China

**Keywords:** DNA methylation, Drought resistance, Reactive oxygen species, Glutathione metabolism, Epigenetic regulation

## Abstract

As an important food crop worldwide, maize is frequently impacted by drought stress. It is of great significance to study its drought resistance genes and mechanisms, in which DNA methylation modification in the promoter region plays a key role in regulating gene expression and stress resistance. In this study, using PEG6000 and 5-azadC to treat different drought-tolerant maize inbred lines (drought-tolerant R99 and drought-sensitive Mo17), combined with transcriptomic analysis, identified the gene *ZmGST2* as being related to drought resistance. Subsequently, the methylation changes in its promoter region were further analyzed. The results indicated that the expression level of *ZmGST2* was closely related to the methylation status of its promoter region under drought stress. Specifically, in drought-resistant R99, a decrease in methylation (demethylation) occurred within the CpG island of the *ZmGST2* promoter region after drought treatment, which facilitated the expression of this gene and thereby enhanced drought resistance in maize. However, in the drought-sensitive Mo17, this demethylation change did not occur, resulting in reduced drought resistance. Further phenotypic analysis revealed that overexpression of *ZmGST2* could enhance the reactive oxygen species scavenging ability in maize roots, improve root growth, and significantly enhance drought resistance. Overall, this study provides both a new candidate gene and a novel approach for molecular breeding aimed at enhancing maize drought resistance.

**Supplementary Information:**

The online version contains supplementary material available at 10.1186/s12870-025-07012-9.

## Background

Maize (*Zea mays* L.), as an important food crop in the world, plays a key role in providing food, feed, and industrial raw materials [1]. However, just like other major food crops, maize growth and productivity is severely challenged by climate change-exacerbated drought stress [2, 3]. Drought not only causes root damage and reduces water absorption but also inhibits photosynthesis and reproductive development, thus significantly reducing yield [4–7]. Therefore, understanding plant responses to drought stress is crucial for developing new cultivars with improved drought tolerance and ensuring food security. Maize responds to drought through a series of physiological mechanisms [8], such as the adaptive growth of roots, leaf curling to reduce leaf transpiration, and the accumulation of osmotic adjustment-related substances such as proline and betaine to maintain cellular water balance [9]. In addition, drought resistance of maize is also regulated at the gene level. Unravelling these different plant stress response mechanisms essentially facilitates the creation of new drought tolerant crop cultivars.

RNA sequencing (RNA-Seq), an advanced technology, can uncover the molecular mechanisms related to drought resistance by analyzing changes in gene expression under drought stress. For example, studies found that *ZmPMP3g* [10], *ZmWRKY79* [11] and other genes play key roles in drought response, which can improve the survival ability of maize in drought environment. Through in-depth study of these drought-resistant genes, scientists can provide theoretical support for the cultivation of drought-resistant maize varieties, and provide effective solutions to cope with the uncertainties brought by future climate change [12]. Therefore, transcriptome sequencing not only provides us with tools to decode the maize drought resistance mechanism, but also provides a new way to improve the drought resistance of maize and ensure food security [13].

Glutathione S-transferase (GST) plays a key role in the plant response to drought stress [14]. Under drought conditions, plants produce a large number of reactive oxygen species (ROS), which can cause oxidative damage and affect plant growth and development. GST promotes ROS removal by binding and transporting toxic substances, thereby alleviating oxidative damage caused by drought and protecting plant cells. For instance, studies have found that GST activity in leaves increases significantly under drought conditions, helping to remove excess ROS and reduce oxidative stress, thereby enhancing the drought resistance of rice [15]. Studies have shown that the expression level of GST in tea trees increases significantly during drought, which can effectively reduce the oxidative damage caused by water stress. In addition to GST, plants also respond to ROS through other antioxidant systems. For example, enzymes such as superoxide dismutase (SOD) and peroxidase (POD) can decompose ROS and reduce cellular oxidative damage [16]. Studies on soybean showed that under drought stress, SOD and POD activities of soybean were also significantly increased, which synergistically acted with GST to enhance the drought-resistant ability of plants [17]. Therefore, GST collaborates with other antioxidant mechanisms, including ascorbic acid and glutathione, to assist plants in coping with stressors such as drought more effectively and in maintaining their growth and physiological functions.

DNA methylation is an important mechanism in plant epigenetics, mainly occurring at the 5-position carbon atom of cytosine in the form of CG, CHG, and CHH sites [18]. In plants, methylation maintains genomic stability through methyltransferases (e.g. MET1, CMT3, CMT2) that inhibit the activity of transposons and foreign DNA [19, 20]. It also regulates the growth and development of plants, such as affecting the vernalization process and plant maturity; abnormal methylation can lead to abnormal development, such as tomato ripening [21]. In addition, DNA methylation plays a key role in plant stress resistance, regulating responses to environmental stresses (such as drought and high temperature), and these methylation changes can sometimes be passed on to offspring, affecting resistance characteristics [22, 23]. Studies have found that plant gene methylation patterns are not only affected by environmental stress, but also transmitted to offspring through heredity to enhance their adaptability. For example, under drought stress, changes in methylation levels may help offspring better cope with similar adversities [24]. Therefore, DNA methylation is not only a mechanism for regulating gene expression but also a key means by which plants cope with environmental changes and maintain genome stability. The DNA methylation inhibitor 5-aza-2'-deoxycytidine (5-azadC) was utilized to study methylation-dependent regulation. As a cytidine analog with a nitrogen substitution at the fifth carbon, it integrates into DNA during replication and irreversibly binds to DNA methyltransferases (DNMTs), inducing global demethylation [25]. This mechanism makes it a powerful tool for identifying genes silenced by methylation. In the study, two materials with varying drought resistance were treated with 20% PEG6000 and 100 μM 5-azadC, respectively, for RNA-Seq analysis. By comparing the changes in gene expression of these two materials under different treatments, the relationship between drought resistance and methylation patterns was further explored. The drought resistance function of the maize gene *ZmGST2* was identified and analyzed using gene silencing techniques and overexpression material construction. Furthermore, the expression differences and function of *ZmGST2* under various drought treatment conditions were investigated. Concurrently, changes in methylation sites within the promoter region were analyzed, and the correlation between these methylation changes and the expression level of *ZmGST2* was examined. The results of this study will provide a theoretical basis and genetic resources for the genetic improvement of maize drought resistance at the seedling stage, and suggest potential molecular targets for enhancing maize’s adaptability to drought conditions.

## Materials and methods

### Plant materials

Maize inbred lines R99, Mo17, and B73 (All these seed materials are sourced from the Corn Germplasm Resources Bank of the Hebei Agricultural University) and *ZmGST2* overexpression (*ZmGST2-*OE) lines (with B104 background) were germinated and grown in the growth room with 16 h light and 8 h dark at 28 ℃.

### Identification of drought resistance in R99 and Mo17

Select the Mo17 and R99 seeds that are plump in size and uniform in shape. Maize seeds were sterilized in 75% (v/v) ethanol for 30 s and washed three times with distilled water. They were then soaked in 1% (v/v) NaClO solution for 10 min, washed with distilled water, placed between two sheets of wet filter paper, and germinated in the dark for 4 d at 28 °C. They were then transferred into a greenhouse with 24/22 ℃ (day/night) temperature and 16 h light/8 h dark cycle conditions. Twenty plants from each line were transplanted into 10 soil pots (10 cm depth × 8 cm diameter: two plants per pot) and grown to three—leaf—one—heart stage. Drought treatment was administered to the plants at the three—leaf—one—heart stage by withholding water until soil moisture content fell to 30% at the initiation (day 0) of drought treatment. Soil moisture content was measured daily (from day 0 to day 12) with a TOR 150 Soil Moisture Meter (Cat# 6435; Spectrum, CA, USA). Twelve days post drought treatment, when a difference in wilting was easily observeable, the plants were rewatered (for drought recovery assessment). The number of surviving plants was recorded 3 d after re-watering. Twelve plants for each line were compared in each survival rate test, and three independent experiments were conducted. Samples were taken from both normal and drought-stricken plants and were rapidly frozen in liquid nitrogen and then stored at −80℃ in a refrigerator. Subsequently, the physiological and biochemical indicators were measured.

### RNA-Seq analysis of Mo17 and R99

The seeds were cultured to two—leaf—one—heart stage in quartz sand after germination and transferred to Hoagland culture medium. Light culture conditions were 16 h light/8 h darkness, humidity was 65%, and temperature was (25 ± 2) ℃. When the maize reached the three—leaf—one—heart stage, the roots were treated with 20% PEG6000 and 100 μmol L^−1^ 5-azadC for 5 days. Subsequently, maize roots with similar growth status were carefully selected, immediately immersed in liquid nitrogen for rapid freezing, and then stored in a—80 ℃ freezer for subsequent experiments. Three biological replicates were set up for each treatment, for a total of 18 samples. The Mo17 and R99 materials that received normal treatment were named as “SC” and “TC” respectively, and those that received drought treatment were named as “SD” and “TD”. The 5-azadC reagent was used for methylation inhibitor treatment in the sensitive and the tolerant inbred lines to create “SI” and “TI” groups, respectively. Raw data (raw reads) in fastq format were subjected to quality control by FastQC (v.0.11.9), and the clean reads were mapped to the reference maize genome (B73 RefGen_v4, AGPv4) using the HISAT2 program (v2.1.0) with default parameters. The read counts of each gene were obtained by Feature Counts (v.2.0.1). Differentially expressed genes (DEGs) were analyzed using the R package DESeq2, (v.1.30.0). *P*-values were adjusted using the Benjamini–Hochberg procedure. DEGs were selected based on the following criteria: *P* < 0.05 and log2 (fold change) ≥ 2. Three independent replicates were performed for each sample at each time point. A Venn diagram analysis was conducted using the online website (https://www.bioinformatics.com.cn). GO enrichment of DEG clusters was performed with the program Phyper (http://www.geneontology.org). The significance of the GO terms was corrected using FDR-values < 0.05. KEGG pathway analysis were carried out by KOBAS 2.1.1 (https://kobas.cbi.pku.edu.cn/download.php) [26].

### Subcellular localization

Combining the key genes identified in published literature and transcriptomics analysis, we selected *ZmGST2* for gene function analysis. The recombinant plasmid pBWA(V) HS-1-GLosgfP-*ZmGST2* was constructed by inserting the coding sequence (CDS) of *ZmGST2* into the *BamH* I/*EcoR* I restriction sites of pBWA(V) HS-1-GLosgfP. The recombinant plasmid and the control vector were transformed into *E. coli* (DH5α) and subsequently introduced into tobacco (*Nicotiana benthamiana*) leaf epidermal cells via Agrobacterium tumefaciens (GV3101) infection. The tobacco used was sourced from the Corn Germplasm Resources Bank of Hebei Agricultural University. The infection solution was injected into the underside of 6-week-old tobacco leaves using a disposable syringe, and the leaves were slowly filled with the bacterial solution. The lower epidermis of tobacco leaves cultured in the dark for 48 h was observed using a FluoViewTM FV1000 Confocal Microscope (OLYMPUS Corporation, Tokyo, Japan) set at 488 nm.

### RT-qPCR analysis

To measure gene expression in maize roots under drought stress, three—leaf—one—heart stage maize (R99) plants were utilized. Root samples were collected daily at the same time, starting from day 0 (the initiation of drought treatment, when soil moisture content was at 20%) and extending until day 8. To explore tissue-specific gene expression patterns, total RNA was extracted from the leaves, stems, and roots of R99 plants. Three plants were pooled for each tissue sample, immediately immersed in liquid nitrogen, and then stored in an ultra-low temperature freezer at −80 ℃. Total RNA was extracted using the versatile Plant RNA Extraction Kit (OminiPlant RNA Kit, Beijing ComWin Biotech Co. Ltd, Beijing, China). cDNA from the total RNA was synthesized using EasyQuick RT MasterMix Kit (Beijing ComWin Biotech). RT-qPCR was conducted on a Roche Light Cycler 96 real-time PCR thermocycler using 2 × Fast Super EvaGreen® qPCR Mastermix (US Everbright Inc, CA, USA). Maize *GAPDH* gene was used as the internal control, and relative gene expression levels were calculated using the 2^−∆∆CT^ method [27]. Primer sequences are detailed in Table S1. The analyses were performed with three biological and three technical replicates.

### Function analysis of *ZmGST2*

For amplification, the coding DNA sequence (CDS) of *ZmGST2* from the cDNA of maize inbred line B73 was ligated into the pBWA(V)BS vector. The recombinant positive plasmid pUbi:: *ZmGST2* was transformed into *Agrobacterium tumefaciens* strain EHA105. Immature embryos of the common maize inbred line B104 were transformed with the assistance of EDGENE BIOT Company (Wuhan, Hubei, China). Multiple transgenic plants were obtained, and three homozygous lines with relatively high expression levels were selected. Additionally, using B73 as the genetic background, we employed Pr CMV-mediated gene silencing to knock down the expression of *ZmGST2*. After 10 days, samples were taken for quantitative reverse transcription polymerase chain reaction (RT-qPCR) to detect the expression of *ZmGST2* in Pr CMV-*ZmGST2* and Pr CMV-*00* (as a control) transgenic plants using specific primers. All these primers and test results used for detection are shown in Table S1 and Figures S2-S3. Plants were grown in plastic boxes (10 cm depth × 8 cm diameter) filled with soil mixture: vermiculite ratio (1:3). Each line (2 plants per box) were planted at 16 h light/8 h dark at 28 ℃.

### Physiological and biochemical indices estimation

Proline (PRO) content, malondialdehyde (MDA) content, Hypohalite peroxide (H_2_O_2_) content, Superoxide anion (O^2−^) content, reduced glutathione (GSH) content and activities of the antioxidant enzymes superoxide dismutase (SOD), peroxidase (POD), glutathione-S-transferase (GST) and catalase (CAT) were determined in drought-stressed and control plants. POD, SOD and CAT activities were quantified following Azevedo et al. [28] and Aebi [29], respectively. Proline content of drought-stressed and control plants was measured by the ninhydrin protocol of Bates et al. [30]. MDA content, as an indicator of the level of lipid peroxidation, was determined as previously described [31]. Hydrogen peroxide content of drought-stressed and control plants was measured by following Zhou et al. [32]. GSH content of drought-stressed and control plants was measured by following Ellman [33]. GST activities were quantified following Habig et al. [34]. Superoxide anion content of drought-stressed and control plants was measured by Ponti et al. [35]. These physiological assays were determined using analytical kits supplied by Suzhou Grace Biotechnology Co., Ltd. (Suzhou, Jiangsu, China), with each experiment or assay replicated three times.

### Root traits measurement and evaluation

Root phenotypes of the three—leaf—one—heart stage maize plants were measured: root length (RL), total root projection area (TRPA), total root surface area (TRSA), total root volume (TRV), root fresh weight (RFW), and root dry weight (RDW). RL was measured using a ruler. The traits TRPA, TRSA and TRV were scanned with Epson Perfection V800 photo and analyzed using the EPSON Scan software (Epson, Nagano, Japan). RL refers to the length of the main root in a plant. RFW of each maize plant was recorded. A pooled sample of 20 roots underwent oven drying. First, it was dried at 105 ℃ for 10 min, and then dried at 65 ℃ until a stable mass was reached to guarantee thorough dehydration. At least three biological and three technical replicates were recorded for each trait.

### Analysis of promoter methylation patterns

CTAB method was used to extract high quality genomic DNA from maize tissues to ensure the integrity and purity of the sample [36]. For the *ZmGST2* promoter region, the 2000 base-pair region upstream of the transcription start site (TSS) was identified by genomic database, and specific primers were designed for amplification. Bisulfite treatment was performed using the EpiArt DNA Methylation Bisulfite Kit, and unmethylated cytosines were converted to uracil, while methylated cytosines remained unchanged. The treated DNA was used as a template for PCR amplification using specific primers and for Sanger sequencing (Shenggong Biotechnology Co., Ltd). By comparing the sequencing data with the reference genome, the methylation status of each CpG site was analyzed, with unconverted cytosine representing methylation and converted cytosine representing unmethylation. Finally, statistical analysis of the data was performed to compare the methylation differences among different samples and find potential methylation markers to further reveal the methylation pattern of the promoter region and its role in biological processes. Primer sequences are listed in Table S1.

### Bioinformatics analysis

For identifying cis-acting regulatory elements in the *ZmGST2* promoter, PlantCARE database (http://bioinformatics.psb.ugent.be/webtools/plantcare/html/) was used to predict promoter sequences within 2000 bp upstream of the 5’UTR. MethPrimer (http://www.urogene.org/methprimer/) was used to predict CpG islands in *ZmGST2*’s promoter. CyMATE (http://www.cymate.org) was used to analyze DNA methylation sites in the promoter region. CyMATE analyzes the sequence data obtained from the genome sequencing of sulfite, and can distinguish the methylation of CG, CHG and CHH (where H = A, C or T). It can also extract quantitative and qualitative data on the general and pattern-specific methylation of each sequence and each position, that is, the data of individual sites in the sequence and the epigenetic differences in the sample [37].

## Results

### Identification of drought resistance in R99 and Mo17

**Fig. 1:**
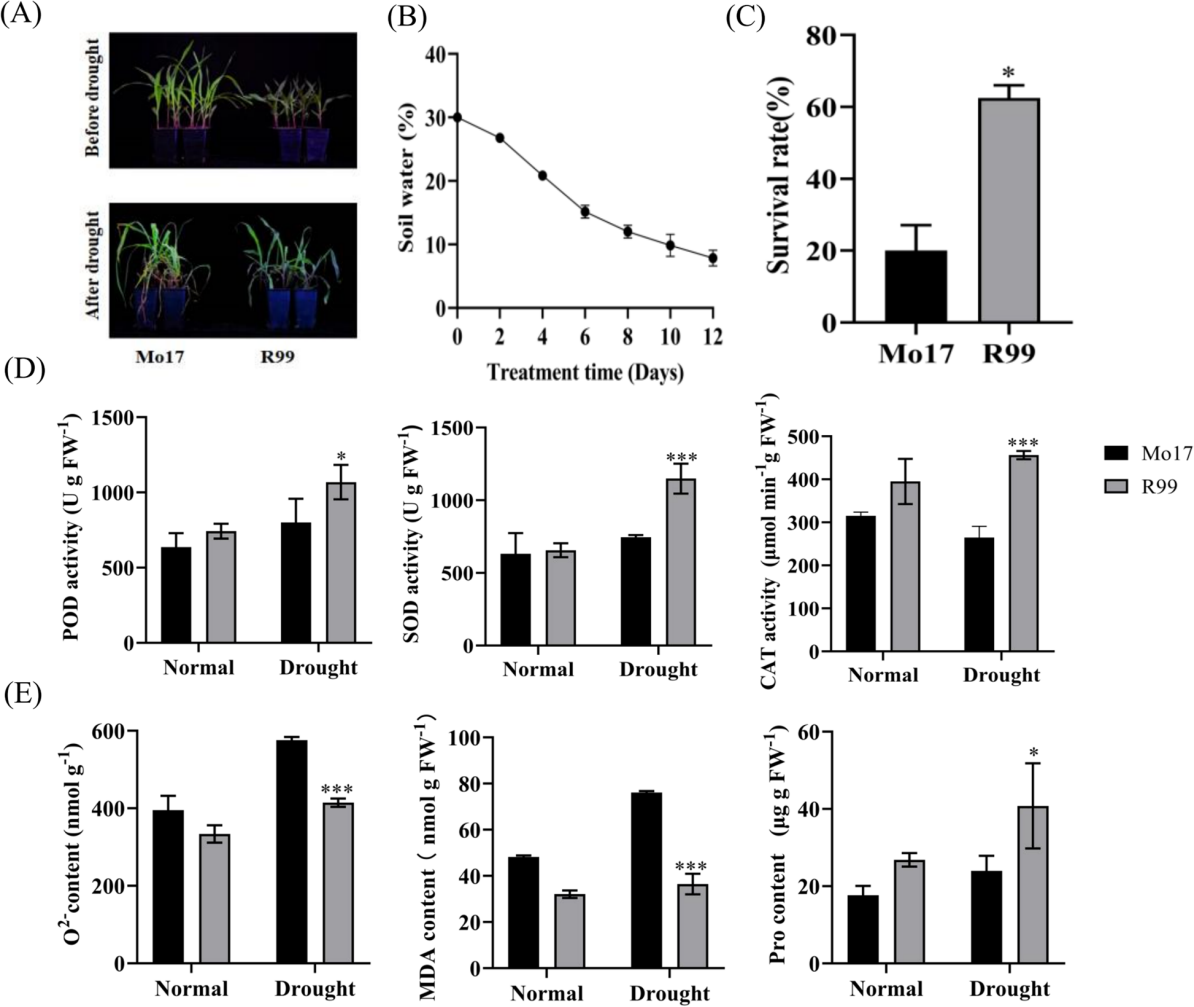
Phenotypes and physiological indices of R99 and Mo17 under normal and drought stress conditions at the seedling stage. **A** Drought stress phenotypes of Mo17 and R99 at the seedling stage. **B** Soil moisture in the pots exposed to drought treatment at the indicated time points. Values are means ± SD (*n* = 3). **C** Survival rate of Mo17 and R99 (*n* = 12). **D** POD, SOD, and CAT activity. **E** O^2−^, MDA, and Pro contents. Error line denotes mean ± SD, the experiment used three biological replicates, (the same as below). Asterisks indicate significant differences compared with Mo17 (*p < 0.05, ***p < 0.001, t-test)

Under normal conditions, R99 and Mo17 exhibited similar growth and leaf development, maintaining normal growth states aside from their inherent varietal differences (Fig. 1A). As the soil water content decreased (Fig. 1B), a significant difference became apparent between Mo17 and R99 when the soil water content reached 0%. The wilting of Mo17 leaves was substantially more severe than that of R99, resulting in visibly withered and yellowed leaves (Fig. 1A, B). Following re-watering, the survival rate was measured, revealing a rate of 62% for R99 compared to 20% for Mo17; thus, the survival rate of Mo17 was significantly lower than that of R99 (Fig. 1C). The activities of catalase (CAT), superoxide dismutase (SOD), and peroxidase (POD), as well as the levels of superoxide anion, malondialdehyde, and proline, were measured in both groups under normal and drought conditions. Under normal conditions, no significant differences were observed in these indices, all remaining within normal ranges. However, under drought stress, R99 exhibited significantly higher activities of CAT, SOD, and POD compared to Mo17, by 41.98%, 35.05%, and 25.11%, respectively (Fig. 1D). These antioxidant enzymes play an important role in plant drought stress resistance. Concurrently, under drought, the levels of superoxide anion and malondialdehyde were 28.01% and 57.85% higher, respectively, in Mo17 than in R99. Both can cause some damage to the plant (Fig. 1E). Both O^2−^ and MDA can cause damage to plant cells. In contrast, the proline content was 41.25% higher in R99 than in Mo17, which helps protect plants from drought stress-induced damage (Fig. 1E). All the measured indices showed significant differences between the two genotypes. These results collectively indicate that R99 is a drought-tolerant material while Mo17 is a drought-sensitive material.

### RNA-Seq analysis results of Mo17 and R99

FPKM (Fragments Per Kilobase per Million reads) values were calculated using the expression quantification software RSEM. Using TPM (Transcripts Per Million) ≥ 1 as the threshold, FPKM values were obtained under normal watering (CK), drought treatment (D), and methylation inhibitor treatment (I) conditions. A total of 8,470 differentially expressed genes (DEGs) were detected between the treatment groups of the drought-tolerant inbred line R99 and the drought-sensitive inbred line Mo17. The sensitive line Mo17 exhibited stronger transcriptional changes under drought stress (6,560 DEGs; 3,854 downregulated, 2,706 upregulated) compared to treatment with the methylation inhibitor 5-azadC (313 DEGs). In contrast, the tolerant line R99 showed fewer drought-induced DEGs (1,514 total; 1,087 upregulated, 427 downregulated) but greater sensitivity to the methylation inhibitor (83 DEGs with 5-azadC). These patterns suggest widespread transcriptional suppression in Mo17 under drought, active stress adaptation in R99 (characterized by preferential upregulation), and stronger epigenetic regulation in R99. This justifies our subsequent focus on *ZmGST2*.

**Fig. 2:**
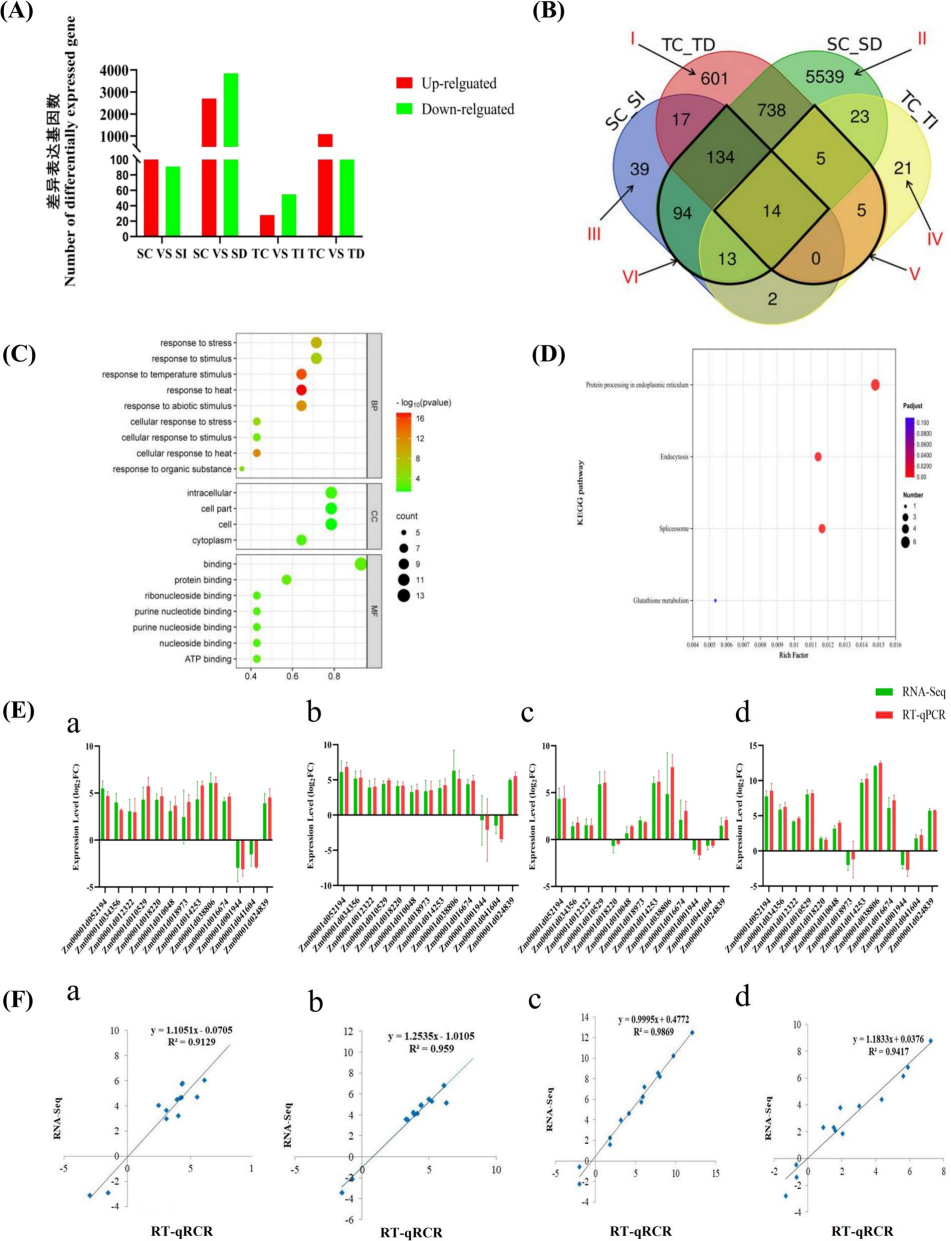
Transcriptome analysis and KEGG metabolic pathway enrichment analysis of DEGs. **A** Statistical chart of DEGs in various comparison group. **B** Venn diagram analysis of DEGs. The overlap region of Venn diagram indicates DEGs overlap between corresponding groups. I, II, III, IV, V and VI respectively represent DEGs in different groups. Sensitive inbred line Mo17 (Sensitive, S), drought resistant inbred line R99 (Tolerant, T), control-C, drought treatment-D, methylation inhibitor treatment-I. **C** The horizontal coordinate is the percentage of genes or transcripts in this secondary classification, and the left vertical coordinate is the secondary classification term for GO, where the greater the -log10 (*P* value) value, the more significant the GO enrichment. The dot size represents the number of genes that are enriched, and the more genes there are, the larger the dot. The right ordinate represents the three categories, BP: Biological process; CC: cell component; MF: Molecular function. **D** The ordinate represents the enrichment results of the KEGG pathway, and the abscess is the ratio of the number of genes enriched into the pathway from the selected gene set to the number of genes enriched into the pathway from the background gene. The greater the -log10 (*P* value) value, the more significant the KEGG enrichment. The dot size represents the number of genes that are enriched, and the more genes there are, the larger the dot. **E** Validation of DEG expression patterns in maize inbred lines Mo17 (drought-sensitive) and R99 (drought-tolerant) under different treatments. (**a**) SC-SD: Mo17 control vs 20% PEG6000; (**b**) SC-SI: Mo17 control vs 100 μM 5-azadC; (**c**) TC-TD: R99 control vs 20% PEG6000; (**d**) TC-TI: R99 control vs 100 μM 5-azadC. Data show log2 fold-changes (RNA-Seq in green, RT-qPCR in red) with *GAPDH* as internal control (error bars: ± SD, *n* = 3). **F** Correlation analysis between RNA-Seq and RT-qPCR data. (a) SC-SD (*R*^2^ = 0.9129); (**b**) SC-SI (*R*^2^ = 0.9590); (c) TC-TD (*R*^2^ = 0.9869); (d) TC-TI (*R*^2^ = 0.9417). Axes show log2-transformed expression values (x: RT-qPCR; y: RNA-Seq), with dashed line indicating perfect correlation (y = x)

DEGs were classified as up-regulated or down-regulated across different treatment groups (Fig. 2A). Among them, for the sensitive variety Mo17, there were 313 differentially expressed genes under the SC and SI treatment conditions (SC_SI), including 222 up-regulated genes and 91 down-regulated genes. Under the SC and SD treatment conditions (SC_SD), there were 6,560 differentially expressed genes, including 2,706 up-regulated genes and 3,854 down-regulated genes. For the drought-resistant inbred line R99, there were 83 differentially expressed genes under the TC and TI treatment conditions (TC_TI), including 28 up-regulated genes and 55 down-regulated genes. Under the TC and TD treatment conditions (TC_TD), there were 1,514 differentially expressed genes, including 1,087 up-regulated genes and 427 down-regulated genes.

Venn diagram was drawn according to the groups for comparative analysis (Fig. 2B), in which 601 and 5539 were the number of drought stress-responsive genes in drought-resistant inbred line R99 and sensitive inbred line Mo17, respectively (Fig. 2B I, II). Region III represented 39 genes specific to SC_SI, of which 9 were down-regulated and 30 up-regulated (Fig. 2B III). Region IV represents 21 genes specific to TC_TI, among which 16 were down-regulated and 5 up-regulated (Fig. 2B IV). PEG6000 treatment and 5-azadC treatment of drought-resistant inbred line R99 and sensitive inbred line Mo17 shared 24 and 255 DEGs, respectively (Fig. 2B V, VI), and there were 14 DEGs between them.

By real-time quantitative assay (RT-qPCR), 13 gene samples were randomly selected from the transcriptome, and their abundance differential expression under PEG6000 and 5-azadC treatment was detected in sensitive and drought-resistant inbred lines, respectively (Fig. 2E). The transcription patterns and levels of 13 genes were consistent with the sequencing results (Fig. 2F). The correlation coefficients (R^2^) were 91.29%, 95.9%, 98.69% and 94.17%, respectively, indicating the reliability of transcriptome data.

A total of 14 DEGs were present in the four test groups (Fig. 2B), and these genes regulate drought resistance through methylation in both varieties. GO annotation analysis was conducted on 14 DEGs (Fig. 2C). According to the above enrichment, it was found that the genes in these enriched items were key genes regulated by methylation in response to drought stress, among which the items with significant enrichment included “response to stress”, “response to stimulus”, “response to temperature stimulus”, “response to heat” and “response to abiotic stimulus”.

KEGG enrichment analysis was performed on genes (Fig. 2D), and the enriched metabolic pathways were “Protein processing in Protein processing in endoplasmic reticulum” (KO04141), “Endocytosis” (KO04144), “Spliceosome” (KO03040), and “Glutathione metabolism” (KO00480). Glutathione metabolism plays a role in the pathway of plant resistance to drought stress [38], and in this study, only *ZmGST2* gene was significantly enriched in glutathione metabolism pathway (KO00480). Therefore, this gene responds to drought stress through methylation, thus, warranting the study of its function.

### Cloning and expression analysis of *ZmGST2*

**Fig. 3:**
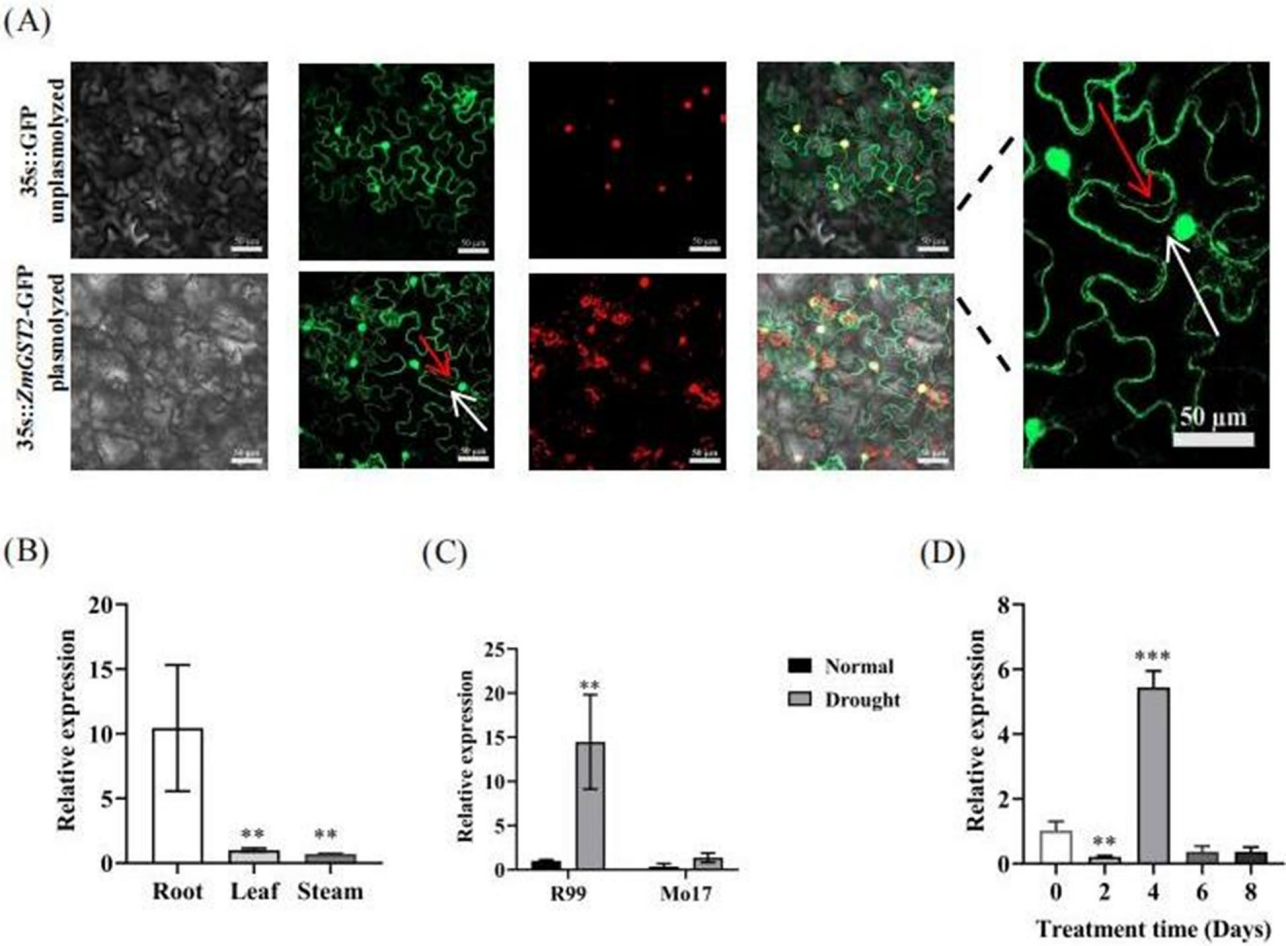
Subcellular localization and tissue-specific expression analysis of *ZmGST2*. **A** Results of fluorescence localization of GFP in the lower epidermis of tobacco leaves. **B** Tissue specific expression analysis of *ZmGST2*. The *GAPDH* was used as the internal reference gene. Asterisk indicate significant differences compared with the control. **C** The expression level of *ZmGST2* in roots of R99 and Mo17 under normal and drought conditions. **D** Expression pattern of *ZmGST2* under drought stress. Asterisk indicate significant differences compared with control (**p < 0.01, ***p < 0.001, one-way ANOVA)

Using CDS sequences of maize inbred lines R99 and Mo17 as the template, the *ZmGST2* coding region sequence was successfully amplified, with a total length of 672 base pairs (bp) (Figure S1A). Upon sequencing comparison, it was determined that the CDS sequencing results for *ZmGST2* were consistent across the two inbred lines (Figure S1B). The total length of the open reading frame of *ZmGST2* gene is 934 bp, with 3 exons and 2 introns (Figure S1C), encoding 224 amino acids. Its protein molecular weight is 24,570.41 Da, the number of primary electrons is 3473, the isoelectric point is 5.76 pI, and the instability coefficient is 53.78. In order to verify the transcriptional self-activation activity of *ZmGST2* gene, subcellular localization was first carried out, and the pBWA (V) HS-1-GloSGFP-*ZmGST2* expression vector was transformed with no-load tobacco by *Agrobacterium tumefaciens*-mediated transient transformation. The lower epidermis of tobacco plant leaves cultured in the dark for 2 days were observed. The results showed that GFP signal of recombinant plasmid pBWA (V) HS-1-GloSGFP-*ZmGST2* existed on the cell wall, plasma membrane and nucleus (Fig. 3A).

The relative expression level of *ZmGST2* gene in the roots, stems and leaves of R99 seedlings was measured, and the expression level in roots was 90.35% and 93.19% higher than that in leaves and stems, both of which were significant (Fig. 3B). Therefore, root-related phenotypes were mainly observed in the identification of drought resistance phenotypes. The relative expression levels of *ZmGST2* in the roots of R99 and Mo17 were measured both before and after drought. Under normal conditions, no significant difference in the expression levels of the two materials was observed (Fig. 3C). However, the expression levels of *ZmGST2* in R99 were significantly higher under drought conditions compared to normal conditions. In contrast, there was no significant change in *ZmGST2* expression in Mo17 before and after drought (Fig. 3C). R99 was subjected to drought stress treatment, and the expression level of this gene in R99 roots was detected on days 0, 2, 4, 6 and 8 (Fig. 3D). The expression level decreased significantly on day 2, reached the highest level on day 4, and decreased on day 6 and day 8. Therefore, when the plant was stressed, it did not respond to drought at the initial stage, and when the drought stress lasted for a long time, the expression of this gene increased significantly to actively respond to drought stress. However, with the aggravation of drought stress, gene expression decreased gradually. Therefore, when plants were subjected to drought stress, the expression of this gene varied with the exposure time period, in response to drought stress.

### Overexpression of *ZmGST2* improved drought resistance of maize

**Fig. 4:**
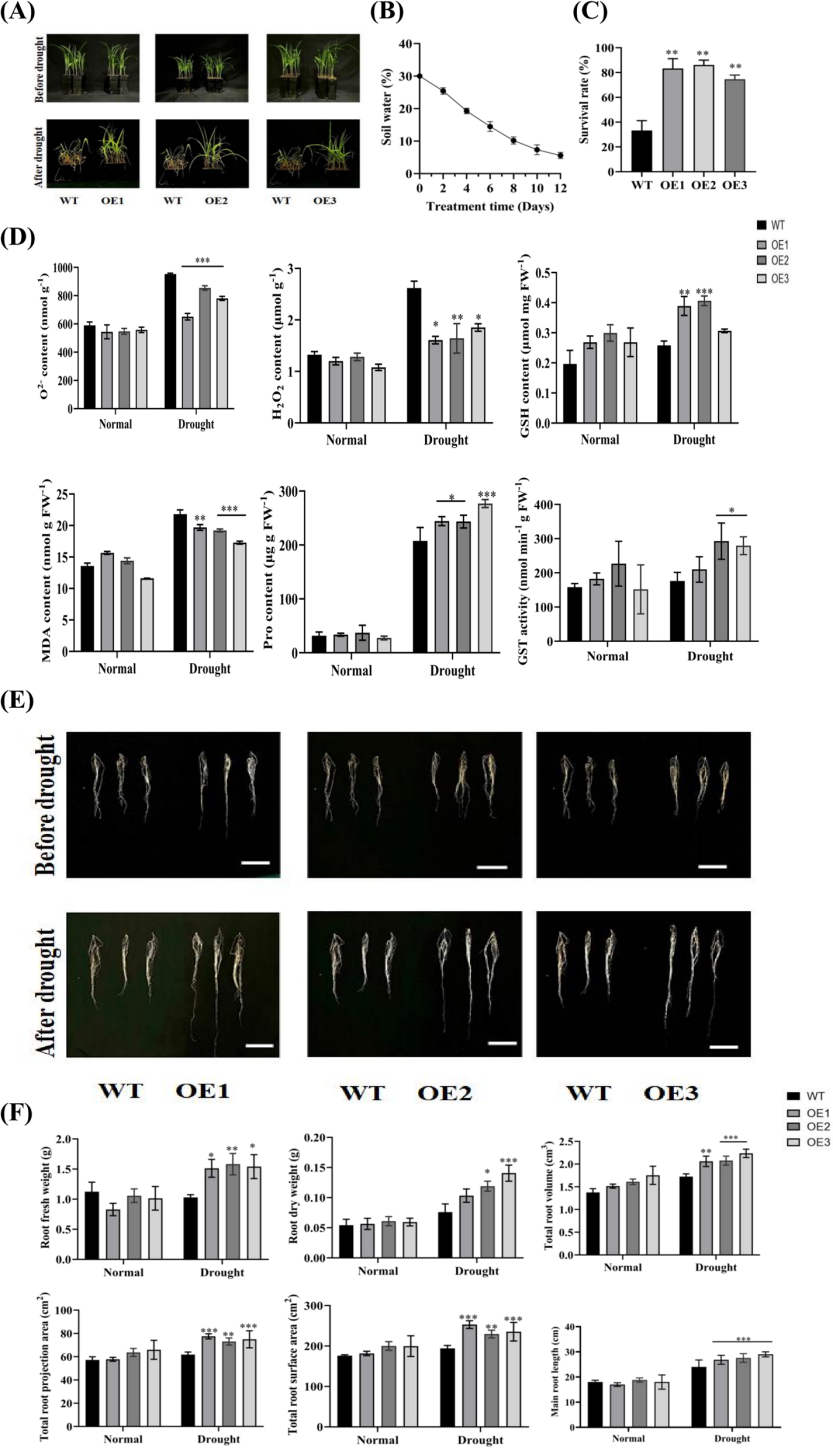
Seedling phenotypes and physiological parameters of WT and *ZmGST2-OE* maize under normal and drought stress conditions. **A** WT and OE1, OE2, OE3 phenotypes under normal and drought conditions. **B** Soil water content (*n* = 3). **C** Survival rates of WT, OE1, OE2 and OE3 under drought conditions. (*n* = 12). **D** O^2−^, H_2_O_2_, GSH content. MDA, Pro content and GST activity. Asterisk indicate significant differences compared with WT. **E** Root morphology of WT and *ZmGST2*-OE before and after drought, scale: 10 cm. **F** Root-related phenotype analysis of WT and *ZmGST2-*OE before and after drought. Asterisk indicate significant differences compared with WT (*p < 0.05, **p < 0.01, ***p < 0.001, one-way ANOVA)

To understand how *ZmGST2* affects maize morphology and drought tolerance, we used RT-qPCR to identify three overexpression lines (OE1, OE2 and OE3) with relatively high levels of *ZmGST2* expression and analyzed their phenotypic, growth status, and physiological responses to drought treatments exposure. Under normal conditions, there was no difference between WT and overexpression lines (Fig. 4A; Figure S2). However, with the decrease of soil water content, there was a significant difference between them when the soil water content reached 0% (Fig. 4B). Compared with overexpression lines, WT had a higher degree of leaf wilting and more serious leaf curling under drought conditions (Fig. 4A), which seriously affected plant growth. As a result, the survival rate of WT was significantly different from that of overexpression lines (Fig. 4C). The results indicated that overexpressed *ZmGST2* could enhance the ability of maize to resist drought stress, reduce the damage caused by drought, and ultimately improve the plant survival rate.

The contents of O^2−^, H_2_O_2_, GSH, MDA, Pro and GST activity in WT and overexpression lines were determined. Under normal conditions, the GST activity of WT and overexpression lines was not significantly different, similar to the contents of O^2−^, H_2_O_2_, GSH, MDA and Pro. However, under drought conditions, the O^2−^ content of WT was higher than that of OE, and the H_2_O_2_ content was higher than that of OE (Fig. 4D), showing significant differences. This indicated that WT accumulated more ROS after drought stress, which had a certain effect on the growth of maize plants. However, in *ZmGST2* overexpression maize lines, the content of ROS was significantly lower than that of WT under drought stress, and the damage to cells and cell membranes was less than that of WT. Meanwhile, the GSH content in WT was reduced compared with OE (Fig. 4D). Pro content decreased by 14.79%−25.06%, GST activity decreased by 16.22%−39.90%, and MDA content increased by 9.63%−20.74% (Fig. 4D). The above data indicated that overexpression of *ZmGST2* could improve the ability of maize to quench ROS, reduce the accumulation of harmful substances, better protect the growth of plant cells, and ultimately improve the drought resistance of maize.

The analysis and photography of the roots of wild-type (WT) and overexpression (OE) lines before and after drought exposure revealed that there was no significant difference in root phenotypes between WT and OE under normal conditions. However, significant differences emerged under drought stress. The roots of WT could not fully absorb soil water during drought conditions, leading to weak root growth and shorter root length (Fig. 4E). Nevertheless, the roots of the overexpression lines remained unaffected by drought conditions and actively responded to drought stress, promoting root growth and water absorption, thereby sustaining above-ground growth. Notably, the fresh weight and dry weight of OE1, OE2, and OE3 increased by 31.94%−34.92% and 12.26%−35.54%, respectively, compared to the wild type (WT) under drought conditions. Meanwhile, the root morphological data indicated no difference between the two materials under normal conditions. Conversely, compared with WT, the total root volume, projected root area, total root surface area and taproot length of OE1, OE2 and OE3 under drought conditions were 31.94%−34.92%, 14.39%−22.24%, 15.46%−23.30% and 16.15%−22.41% higher, respectively (Fig. 4F). The above data indicate that *ZmGST2* regulates drought resistance of maize by affecting root growth. Overexpression of *ZmGST2* is beneficial to root growth, and maize can absorb more water and minimize drought-induced harm.

### Silencing *ZmGST2* reduces drought tolerance in maize

**Fig. 5:**
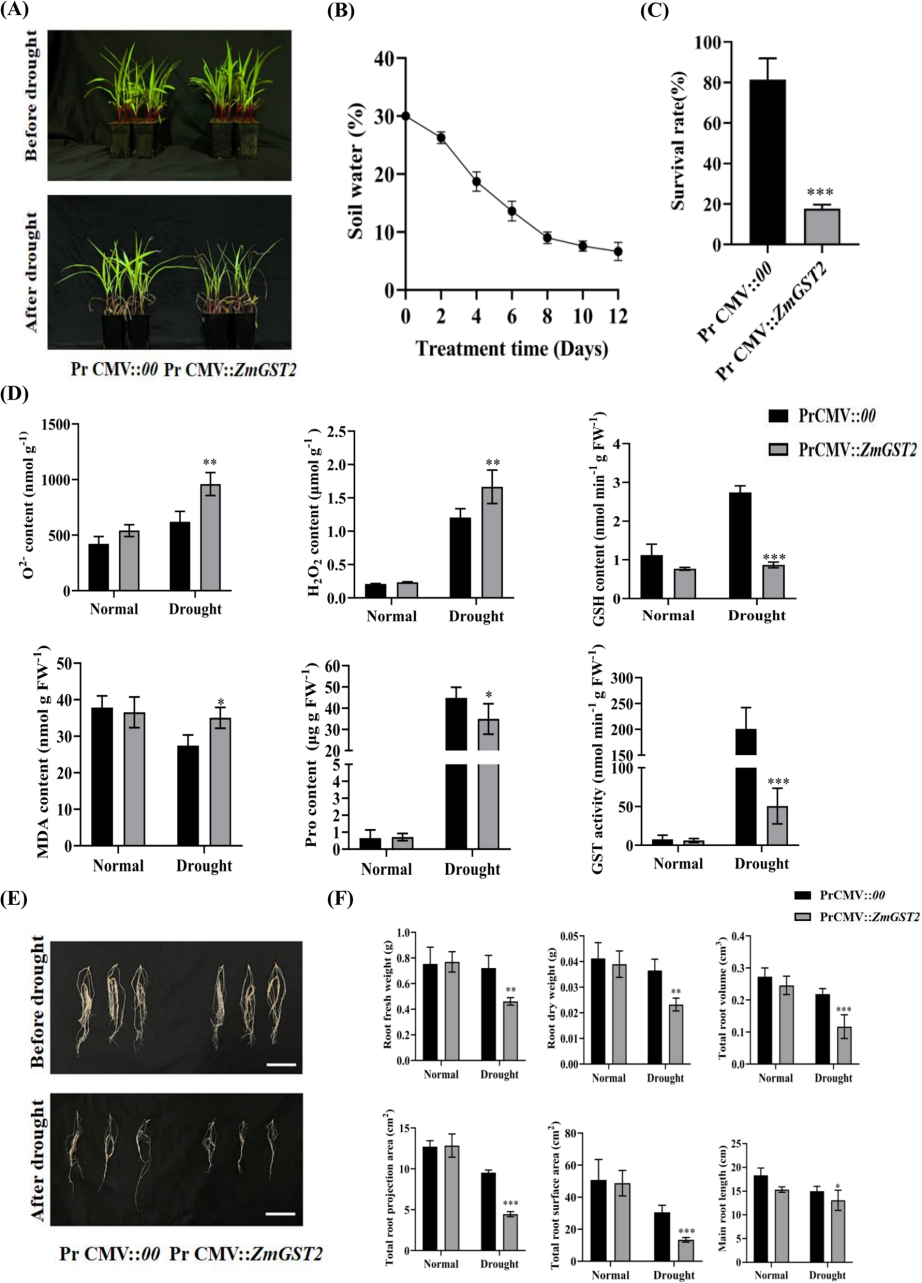
Silencing *ZmGST2* decreased drought tolerance of maize. **A** Pr CMV::*ZmGST2* and Pr CMV::*00* normal and arid phenotypes. **B** Soil water content (*n* = 3). **C** Survival rates of Pr CMV::*ZmGST2* and Pr CMV::*00* under drought conditions (*n* = 12). **D** O^2−^, H_2_O_2_, GSH, MDA, Pro content and GST activity. **E** Root morphology of Pr CMV:*:00* and Pr CMV::*ZmGST2* before and after drought. The scale bar represents 10 cm. **F** Root-related phenotype analysis of Pr CMV::*ZmGST2* and Pr CMV::*00* before and after drought. Asterisk indicate significant differences compared with Pr CMV::*00* (*p < 0.05, **p < 0.01, ***p < 0.001, t-test)

Further, we used VIGS method to generate *ZmGST2*-silenced line, *zmgst2*, and subjected *zmgst2* and WT to natural drought treatment, after which we analyzed their phenotypic and physiological responses (Figure S3). Under normal conditions, there was no significant difference between Pr CMV::*ZmGST2* and Pr CMV::*00* (Fig. 5A). Drought treatment was initiated when the WT and *zmgst2* plants had reached three—leaf—one—heart stage. With the decrease of soil water content (Fig. 5B), differences in WT and *zmgst2* plants became significant when the soil water content reached 0%. Compared with Pr CMV::*00*, the degree of leaf wilting and leaf curling were more severe in Pr CMV::*ZmGST2* (Fig. 5A). After rehydration, the survival rate of the normal material was 63.66% higher than that of the silenced material (Fig. 5C), suggesting that silencing the *ZmGST2* could decrease the maize drought resistance at the seedling stage.

The contents of O^2−^, H_2_O_2_, GSH, MDA, Pro and GST activity in normal and *ZmGST2*-silenced plants were determined. Under normal conditions, the contents of O^2−^, GSH, MDA and Pro and the activities of glutathione transferase were not different between the two materials. However, under drought conditions, the content of O^2−^ and H_2_O_2_ in the silenced materials was 35.31% higher than that in the normal materials, and the content of H_2_O_2_ in the silenced materials was 27.71% higher than that in the normal materials, indicating that the silenced materials accumulated more ROS. It is most probable that ROS damaged plant cell membranes, resulting in increased membrane lipid peroxidation, cell death and loss of function, consequently reducing the drought resistance of *ZmGST2*-silenced materials (Fig. 5D). MDA causes certain harm to cell membrane and cells, and is one of the important indicators to evaluate the degree of cell damage [39]. Thus, the content of MDA accumulation in silent materials was 21.66% higher than that in normal materials (Fig. 5D). GSH is an important antioxidant enzyme for removing ROS and binding toxic substances in plants [40]. Equally, Pro is often accumulated in various plant species in response to different environmental stresses, helping plants survival such harsh conditions [41]. In the current study, Pro content and GST activity were 8.4 % and 74.81 % lower than normal materials (Fig. 5D). The contents of O^2−^, H_2_O_2_, GSH, MDA, Pro, and GST activity were determined under normal and drought conditions. It was found that silencing *zmgst2* had no effect on the normal growth of plants, but it could affect their drought resistance.

After the roots collected from plants subjected to normal and drought conditions were cleaned, photos were taken and analyzed with EPSON scanner. Under normal conditions, there were no significant differences in root phenotypes, root length and growth between normal and *ZmGST2*-silenced materials. However, under drought conditions, the root growth of silenced materials was weaker and the root length was obviously shorter (Fig. 5E). Under drought conditions, normal materials actively extended its roots to absorb water from the deeper soil layers, while *zmgst2* plants accumulated more ROS in roots, causing certain damage to cells and cell membranes, consequently reducing root growth. At the same time, the fresh weight and drought weight of normal materials were 21.85% and 36.30% higher than those of the *ZmGST2*-silenced materials. These results showed that the dry matter content of the root system of the silenced materials was significantly decreased under drought stress conditions. The root morphological data showed that the total root volume, total root projected area, total root surface area, and taproot length of normal materials were 46.55%, 53.22%, 56.15%, and 19.78% higher than those of *ZmGST2*-silenced materials under drought stress, respectively, while there was no difference under normal conditions (Fig. 5F). Overall, these results showed that silencing *ZmGST2* had no effect on root growth under normal conditions, but reduced *ZmGST2*-silenced plants`ROS scavenging capacity under drought conditions, causing damage to cells, making roots unable to fully absorb soil water, affecting root growth and reducing the drought resistance of silenced materials.

### Analysis of the methylation sites of the *ZmGST2* promoter region

**Fig. 6:**
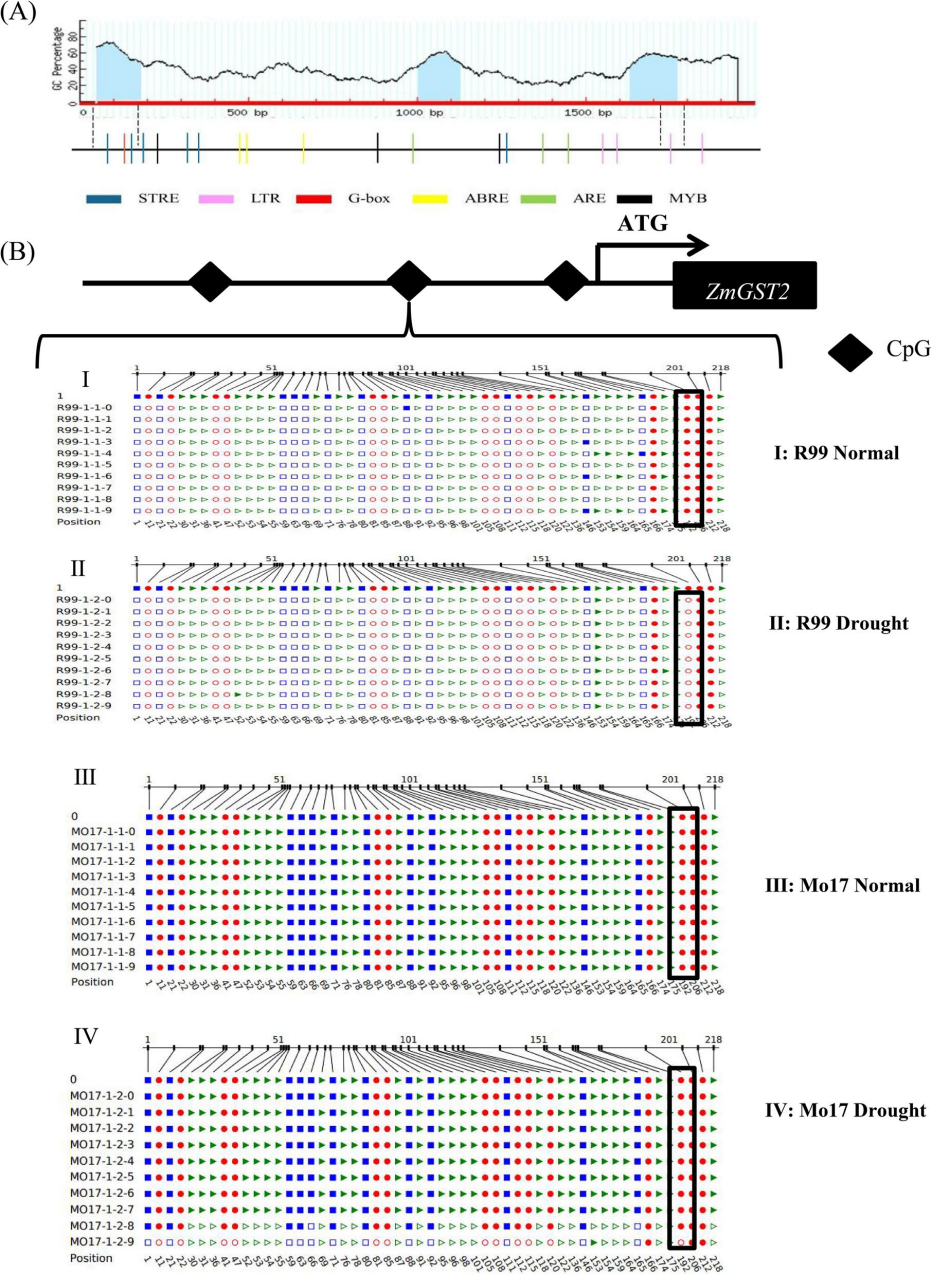
Analysis of DNA methylation sites in *ZmGST2* promoter of contrasting drought-resistance maize inbred lines under normal and drought stress conditions. **A** CpG island structure analysis of *ZmGST2* promoter. (**B**-I) Methylation site analysis in R99 normal materials. (**B**-II) Analysis of methylation sites in R99 drought materials. (**B**-III) Methylation site analysis in Mo17 normal materials. (**B**-IV) Analysis of methylation sites in Mo17 drought materials

The first 2000 bp upstream of the *ZmGST2* gene start site (ATG) were selected as its promoter and cloned in R99 and Mo17, respectively, with a length of 2083 bp. The band length was detected by agarose gel electrophoresis (Figure S4A). Online websites were utilized to analyze the cis-acting elements and CpG islands on the promoter of this gene (Fig. 6A). The three CpG islands were located upstream of the start codon at positions (−48 ~ −183), (−1005 ~ −1128), and (−1633 ~ −1772), respectively. The STRE (−96 ~ −101, −182 ~ −187), G-BOX (−144 ~ −150), and LTR (−1762 ~ −1768) cis-acting elements were located within the first and third CpG islands. Following monoclonal sequencing, it was determined that the sequence of the *ZmGST2* promoter in the two materials was completely identical (Figure S4B).

After the DNA extracted from the two materials was treated with bisulfite and purified, CpG island primers on the promoter of the *ZmGST2* were designed using an online website. A sequence length of 297 bp was successfully cloned from both Mo17 and R99, under normal and drought-stressed conditions. Ten monoclonal tests were performed, and the methylation sites were analyzed using the online website CyMATE compared with the original sequence. In drought-resistant inbred lines, differences were observed at site 192 (highlighted in the black box) between normal and drought conditions (Refer to Fig. 6B I, II). Demethylation occurred at this site in drought-resistant inbred lines under drought conditions. The data reveal a drought-responsive CHH context (where H = A/C/T) demethylation event at −810 bp (relative to the TSS) in the resistant line R99, which correlates with enhanced *ZmGST2* expression. This occurred despite being outside the characterized CpG islands. This suggests that non-CpG methylation plays a functional role in drought adaptation, potentially by modulating transcription factor binding and chromatin accessibility. while no demethylation occurred at site 192 in sensitive inbred lines under normal and drought conditions (Fig. 6B III and B IV). In addition, as shown in Fig. 6B II, *ZmGST2* expression in R99 roots under drought conditions was significantly higher than that under normal conditions. The expression of *ZmGST2* was also higher than that of Mo17 under drought conditions. However, there was no significant difference in gene expression before and after drought in Mo17 material. It was speculated that the change of methylation site affected the expression level of *ZmGST2* in R99, which resulted in a significant difference in drought resistance between the two materials.

## Discussion

Maize is an important food crop also central in animal feed and industrial raw material provision [42]. Demand for its production is expected to double by 2050 [43]. However, maize encounters different kinds of stresses during its growth, including drought, high temperature, waterlogging, low temperature and pests and diseases. Drought is a major meteorological natural disaster with a high frequency, a long duration and the widest scope of damage [44], which threatens maize yield when it is relatively severe. According to statistics, 70% of China’s maize planting area will be affected by drought stress, resulting in annual yield loss of about 1.5 × 10^7^t. Severe drought stress during the seedling growth period often leads to impaired photosynthesis and stunted growth, ultimately affecting maize yield [45]. Therefore, it is of great significance to mine drought resistant genes and cultivate high-yield drought resistant varieties to maintain food demand and sustainable agricultural development [46].

### Mining drought resistance genes through RNA-Seq analysis

In recent years, the application of high-throughput sequencing technology has enhanced the unravelling of mechanisms of plant resistance to various stresses, simultaneously shortening research time. Particularly, RNA-Seq transcriptome analysis technology has become widely used in drought stress response studies [47]. For example, this method was employed to identify nine genes associated with drought stress resistance in the roots of Apophyllum japonicum. Functional verification studies on two of these genes confirmed their critical roles during stress conditions [48]. Transcriptome sequencing analysis of okra under drought stress revealed that the majority of differentially expressed genes (DEGs) were primarily associated with categories such as “membrane components”, “oxidation–reduction process”, and “metal ion binding”, offering a foundation for further insights into the molecular mechanisms of drought resistance [49]. By analyzing the transcriptome sequencing of alfalfa after drought treatment, four transcription factors related to drought were screened. Overexpression of *Mf ERF053* in Arabidopsis increased the root length and lateral root number of transgenic plants. The expression level of stress resistance-related genes was increased, which heightened the sensitivity of crops to drought, salt and ABA [50]. After transcriptome sequencing of 200 wheat materials at seedling stage, differential gene expression analysis was conducted, and some of the genes were involved in water stress and played a role in the biological process of ABA response.

Similar to previous studies, in this study, Mo17 and R99 were treated with 20% PEG6000 and 100 μmol L^−1^ 5-azadC, and a total of 8470 DEGs were identified (Fig. 2A). Among these, 14 DEGs shared between the two inbred lines (as screened by Venn diagram; Fig. 2B) were found to be enriched in “response to drought stress through methylation”. The genes enriched were mainly heat shock proteins (HSPs) and *GST*, both of which have been strongly linked to drought stress response. Hu et al. [51] found that after exogenous ABA application in maize under drought stress, the expression of *HSP70* would increase, thus enhancing the activity of related antioxidant enzymes, enhancing the scavenging of ROS, and improving the drought tolerance of plants. At the same time, overexpression of *OsHSP50.2* in rice increased the proline content in the plants and enhanced drought tolerance [52]. The selected DEGs were enriched (by GO analysis) in maize materials with different drought resistance, and significantly enriched into the glutathione metabolic pathway [53], which is similar to the results of this study. Therefore, glutathione metabolism has research significance in maize drought stress resistance.

### *ZmGST2* clears the reactive oxygen species produced in maize roots under stress

In this study, through transcriptome analysis of maize materials with different drought resistance, we observed that the expression level of *ZmGST2* in the two materials was increased after treatment with PEG6000 and 5-azadC. *ZmGST2* belongs to the Phi class of glutathione transferases, and studies have found that Phi plays an important role in stress resistance, cell signal transduction and disease resistance [54]. It is well known that ROS (Reactive Oxygen Species) are in a stable state within plants, and their content within cells must be strictly regulated. When the homeostasis of ROS is disrupted, they can cause damage to proteins, nucleic acids, and lipids within cells [55]. ROS scavenging systems in plants encompass enzymatic antioxidant and non-enzymatic systems. The antioxidant enzyme system includes SOD, POD, CAT, GPX and GST [55], while the non-enzymatic system includes ascorbate, reduced glutathione, vitamin E, flavonoids and proline, all of which have the ability to clear ROS [56]. In recent years, wheat *TaBZR2* has been directly bound to *TaGST1* promoter to activate its transcription, and *TaGST1* has been shown to clear ROS under drought stress [57]. The content of H_2_O_2_ and superoxide anion in *osgst4* material after drought stress treatment was significantly higher than that in normal materials, which is similar to the results of this study. Here, under normal conditions, there was no significant difference in root ROS content between Pr CMV::*ZmGST2* and Pr CMV::*00*. However, under drought stress conditions, the content of O^2−^ in the *ZmGST2*-silnced material was 35.31% higher than that in the normal material, and the content of H_2_O_2_ in the silences material was 27.71% higher than that in the normal material. Under drought conditions, the O^2−^ content of WT was 10.32%−31.69% higher than that of OE1, OE2 and OE3. The content of H_2_O_2_ was 29.24%−38.57% higher than that of OE1, OE2 and OE3. All these results imply that *ZmGST2* can quench excess ROS produced by roots during drought stress to improve maize drought tolerance.

### *ZmGST2* overexpression enhances maize root growth under drought stress conditions

Here, the *ZmGST2* coding region was successfully cloned. Real-time fluorescence quantification showed that *ZmGST2* expression was significantly higher in roots than in stems and leaves (Fig. 3B). In order to further verify whether this gene is involved in drought stress, R99 was treated with drought for 8 days at the seedling stage, and real-time fluorescence quantification used to analyze its expression. The results showed that *ZmGST2* expression decreased on the 2nd day, significantly increased on the 4th day, and decreased on the 6th and 8th day (Fig. 3D). It is speculated that, on one hand, ROS may act as stress signals in the early stage of drought to enable plants to actively establish a drought defense mechanism. On the other hand, plants inhibit the expression of this gene in order to reduce the clearance of ROS and regulate the transmission of stress signals [58]. With the aggravation of stress, ROS gradually increase, and transcription factors that play a regulatory role may bind to the promoter of the gene [59] or the methylation sites of the promoter may change to activate the expression of the gene, resulting in an increase in its expression level [60]. However, with the aggravation of stress, the drought resistance function gradually weakens. The expression of *ZmLAZ1-3* in different inbred lines under drought stress changed significantly compared with that without drought stress [61], so this gene responds to drought stress and participates in drought stress. *ZmWRKY40* showed a normal distribution of expression after being subjected to drought stress, and this gene responded to drought stress by clearing excess ROS [62]. This is similar to the results of this study, so the next step is to investigate the drought resistance function of this gene.

*OsGSTU6*, a gene contributing to rice resistance to cadmium stress through maintaining ROS homeostasis under stress conditions, exemplifies the diverse functions of GSTs [63]. *AtGSTF8* and *AtGSTU19* maintained redox homeostasis in roots and affected meristem size [64]. Similarly, in this study, related root phenotypes were also affected. Under normal conditions, there was no significant difference between the roots of Pr CMV::*ZmGST2* and Pr CMV::*00*. However, under drought stress conditions, the root growth of Pr CMV::*ZmGST2* was significantly weaker than that of Pr CMV::*00*. We speculated that the plants` ability to remove ROS was reduced, and ROS existed more in the roots, which was not conducive to root growth. In overexpression lines, the root development of *ZmGST2*-OE was better than that of WT, and the content of ROS was significantly lower than that of wild type under drought conditions (Fig. 4E). Studies have shown that maize’s well-developed root system helps it resist drought. *ZmTIP1* plays an active role in regulating maize root hair elongation and drought resistance [65], and *ZmSAUR21* positively regulates H^+^ -ATPase activity and changes cell size in response to auxin, thereby regulating lateral root growth [66]. The overexpression of *ZmDRO1* in maize improved plants` root penetrative ability under drought stress, which consequently enhanced plant soil water extraction and increased yield [67]. In this study, after maize roots were subjected to drought stress, leaf yellowing and wilting appeared, which signified the common drought responses, including reduced photosynthesis, reduced later growth and ultimately affected yield. Nonetheless, *ZmGST2*-OE plants had better root development than the wild type under drought stress, suggesting that overexpression of *ZmGST2* enhances root development in maize under drought stress conditions.

### Demethylation of *ZmGST2* promoter under drought stress promotes *ZmGST2* gene expression and plant drought tolerance

DNA methylation plays an important role in plant growth, development and stress response. Studies have shown that DNA base methylation at different locations (the fifth carbon atom of cytosine and the sixth nitrogen atom of adenine) crucially underpins gene expression regulation [68]. DNA methylation affected the normal growth of Arabidopsis. Mutations in genes that control DNA methylation slowed growth and induced severe developmental deformities, but did not affect the survival time of the plants [69]. *SlALKBH2* transcription is negatively regulated by DNA methylation, and *SlALKBH2* mediates m6A mRNA methylation, thus promoting the mRNA degradation of *SlDML2* and affecting fruit ripening [70]. The methylation of gene promoters is also associated with plant growth and environmental changes. The promoter of the gene may be methylated to activate gene expression, or demethylation may combine with transcriptional activators to promote transcription, which then actuate some genes during plant growth or stress response. For instance, *MdAGO4s* binds to the *MdMYB1* promoter and recruits DNA methylase MdDRM2s to methylate the *MdMYB1* promoter and affect the production of anthocyanin [71].

Studies have shown that DNA methylation status changes under stress [72]. In rice, random polymorphism approach was used to detect methylation status under different water treatment conditions, and methylation sites increased when drought stress intensified [72]. Genes *ZEP31*, *ZEP160* and *ZEP35* in rice respond to stress by increasing or decreasing methylation sites and controlling gene expression under normal and salt stress conditions. A glutathione transferase gene was screened in potato through transcriptomic analysis. The promoter CpG island contained cis-acting elements ABRE and CAAT-box associated with drought, and it was speculated that the response of this gene to stress could be activated by reducing the methylation level in this region [73].

Identical *ZmGST2* promoter and coding region sequences in the two maize materials contrasted with their differing drought resistance, suggesting that changes in promoter methylation sites before and after drought could affect *ZmGST2* expression. After bisulfite sequencing, under normal conditions, the methylation site of *ZmGST2* promoter in R99 material was significantly less than that in Mo17 material, but there was no difference in the relative expression level of *ZmGST2*. After drought treatment, the upper promoter site was demethylated in R99, but did not change in Mo17, and the relative expression of *ZmGST2* was different between the two materials, and the drought-resistant material was significantly higher than the normal material. We speculate that the change of promoter methylation site of this gene leads to the change of its expression level, which leads to the difference in drought resistance between the evaluated maize materials. Under normal conditions, the methylation sites of Mo17 were significantly more than those of R99 materials. We hypothesize that when R99 is under drought stress, the demethylation sites upstream of the promoter of this gene can bind to transcription factors to promote the expression of *ZmGST2*, thereby increasing gene expression and drought resistance of plants. Demethylation and methylation of *ZmEXPB2* promoter occurred under drought conditions, and the relative expression of gene was increased, which promoted the growth of initial roots and enhanced maize drought resistance [74]. This is similar to the results of this study; thus, analyzing the changes of promoter methylation sites is of great significance for mining drought resistance genes.

## Conclusions

In this study, we investigated the function and regulatory role of *ZmGST2* in DNA methylation-mediated drought resistance in maize. Transcriptomic analysis revealed that *ZmGST2* was associated with various biological processes, including glutathione metabolism and redox reactions, and its expression significantly increased following drought treatment. The *ZmGST2* promoter region contains multiple cis-acting elements related to drought response, and the ZmGST2 protein is mainly localized in the nucleus, cell wall and membrane. The experimental results showed that *ZmGST2*-overexpressed lines exhibited higher drought resistance under drought conditions. Additionally, the physiological and growth parameters of their roots, including total root volume and root surface area, were significantly improved compared to the wild type control. Furthermore, analysis of the gene promoter methylation sites revealed that in drought-resistant inbred line R99, *ZmGST2* promoter showed demethylation after drought treatment, while sensitive inbred line Mo17 showed no significant methylation change. In summary, the demethylation of the *ZmGST2* promoter modulates *ZmGST2* gene expression and enhances drought resistance in maize, offering a novel theoretical foundation for the molecular breeding of maize drought tolerance.

## Supplementary Information

Supplementary Material 1: Supplementary Figure 1. Cloning and structural characterization of *ZmGST2* in maize inbred lines. (A) Electrophoresis of PCR-amplified *ZmGST2 *CDS from R99 and Mo17 (M: 2000 bp marker; CK: negative control). (B) Sequence alignment confirming identical CDS regions (672 bp) between R99 and Mo17. (C) Gene structure diagram showing 3 exons (boxes) and 2 introns (lines) spanning 934 bp. Supplementary Figure 2. Expression validation of *ZmGST2*-overexpressing transgenic lines. RT-qPCR analysis showing significantly higher (*P*<0.01) *ZmGST2* transcript levels in three overexpression lines (OE1-3) compared to wild-type (WT) controls. *GAPDH* served as an internal reference (mean ± SD, *n*=3 biological replicates). Supplementary Figure 3. Virus-induced gene silencing (VIGS) of *ZmGST2*. (A) Phenotypic validation showing chlorosis in pCMVZ2-2bN81::*ZmIspH* positive controls (scale: 10 cm). (B) RT-qPCR confirmatio*n of Zm*GST2 silencing efficiency (*P*<0.01) in PrCMV::*ZmGST2* plants versus empty vector controls (PrCMV::*00*). Data represent mean ± SD (*n*=3). Supplementary Figure 4. Promoter cloning and sequence analysis of *ZmGST2*. (A) PCR amplification of 2083 bp promoter regions from R99 and Mo17 (M: 2000 bp marker; CK: negative control). (B) Sequence alignment demonstrating 100% identity between R99 and Mo17 promoter sequences (2000 bp upstream of ATG).

Supplementary Material 2: Table S1. Sequences of primers PCR used in the study.

## Data Availability

The data that support the findings of this study are available from the corresponding author upon reasonable request.
