# Supplementary figures and images for: CHH demethylation in the ZmGST2 promoter enhances maize drought tolerance by regulating ROS scavenging and root growth
Source: BMC Plant Biol. 2025 Aug 18;25:1083. doi: 10.1186/s12870-025-07012-9 (PMC12359901; doi:10.1186/s12870-025-07012-9)

(A)

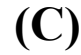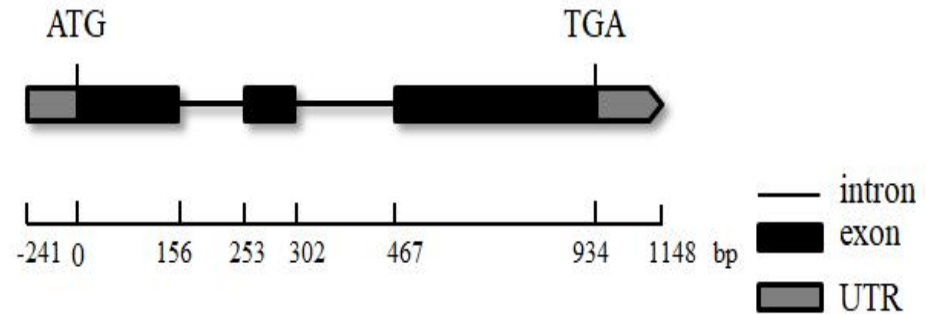

**(B)**

[illegible]

Fig. S2

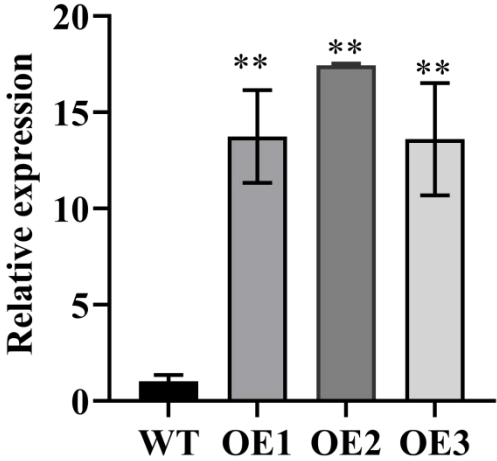

Fig. S3

(A)

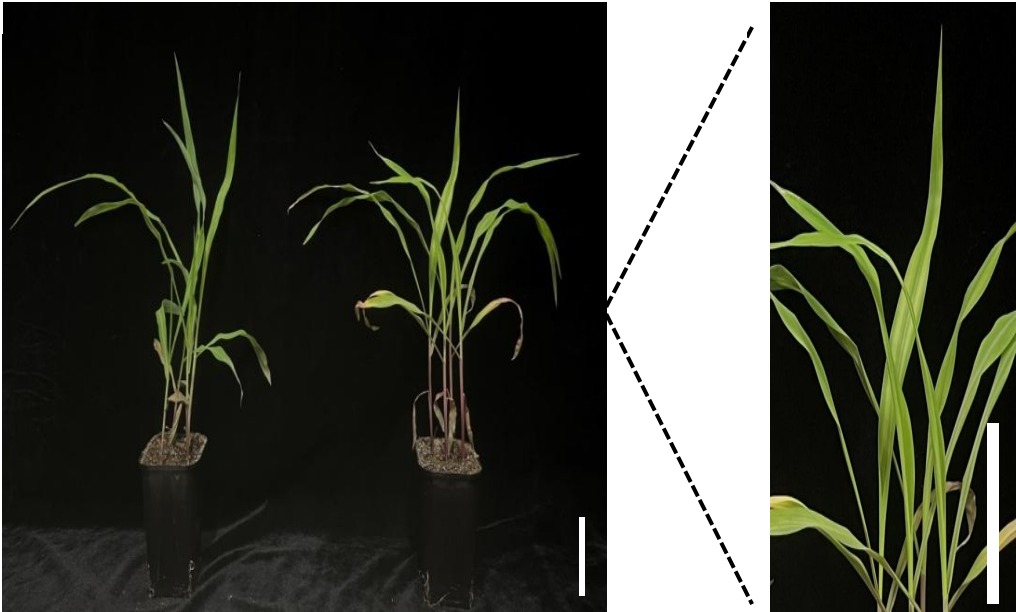

Pr CMV::00    Pr CMV:: *ZmIspH*

(B)

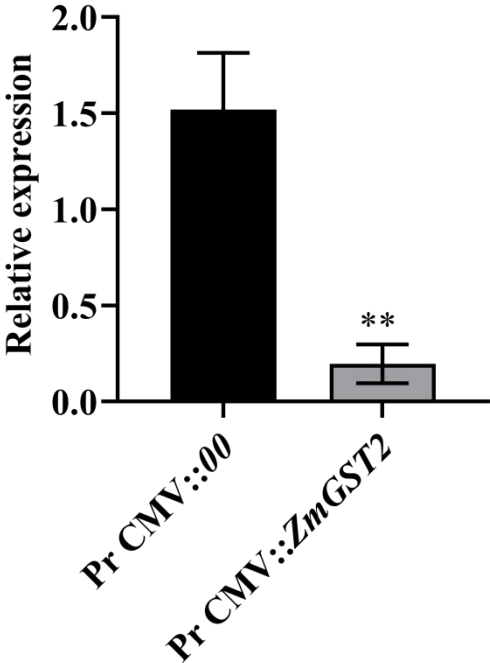

**(B)**

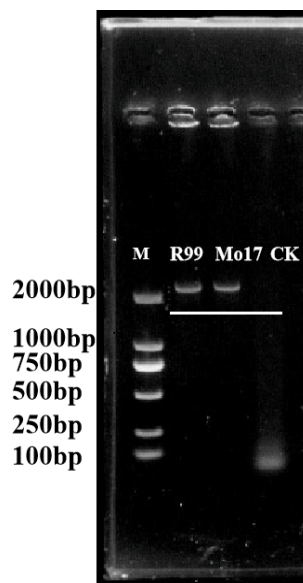[illegible]

Supplement: Supplementary file 1 — Supplementary Material 1: Supplementary Figure 1. Cloning and structural characterization of ZmGST2 in maize inbred lines. (A) Electrophoresis of PCR-amplified ZmGST2 CDS from R99 and Mo17 (M: 2000 bp marker; CK: negative control). (B) Sequence alignment confirming identical CDS regions (672 bp) between R99 and Mo17. (C) Gene structure diagram showing 3 exons (boxes) and 2 introns (lines) spanning 934 bp. Supplementary Figure 2. Expression validation of ZmGST2-overexpressing transgenic lines. RT-qPCR analysis showing significantly higher (P<0.01) ZmGST2 transcript levels in three overexpression lines (OE1-3) compared to wild-type (WT) controls. GAPDH served as an internal reference (mean ± SD, n=3 biological replicates). Supplementary Figure 3. Virus-induced gene silencing (VIGS) of ZmGST2. (A) Phenotypic validation showing chlorosis in pCMVZ2-2bN81::ZmIspH positive controls (scale: 10 cm). (B) RT-qPCR confirmation of ZmGST2 silencing efficiency (P<0.01) in PrCMV::ZmGST2 plants versus empty vector controls (PrCMV::00). Data represent mean ± SD (n=3). Supplementary Figure 4. Promoter cloning and sequence analysis of ZmGST2. (A) PCR amplification of 2083 bp promoter regions from R99 and Mo17 (M: 2000 bp marker; CK: negative control). (B) Sequence alignment demonstrating 100% identity between R99 and Mo17 promoter sequences (2000 bp upstream of ATG). [file 12870_2025_7012_MOESM1_ESM.pdf]
